# Inferring accumulation times of mitochondrial DNA deletion mutants from cross-sectional single-cell data: methodological framework and validation

**DOI:** 10.1038/s41514-026-00431-4

**Published:** 2026-06-16

**Authors:** Axel Kowald, Thomas B. L. Kirkwood

**Affiliations:** 1 Rostock University Medical Center, Institute for Biostatistics and Informatics in Medicine and Aging Research (IBIMA), Rostock, Germany; 2 National Innovation Centre for Ageing, Newcastle University, Newcastle upon Tyne, UK

**Keywords:** Computational biology and bioinformatics, Genetics

## Abstract

The accumulation of mitochondrial DNA (mtDNA) deletion mutants in post-mitotic cells is a hallmark of mammalian ageing and a key contributor to tissue decline in skeletal muscle and neurons. A transcription-coupled replication model predicts that deletions affecting a negative feedback mechanism gain a selective replication advantage, leading to relatively short accumulation times for mutant takeover. However, these accumulation times are experimentally inaccessible since single-cell measurements are destructive. Here, we present a framework to infer such accumulation times from cross-sectional single-cell RNA sequencing (scRNAseq) data, exploiting the fact that mtDNA deletions are also reflected at the transcript level. To establish feasibility, we generated synthetic datasets using two stochastic models of the mitochondrial life cycle and used these as a gold standard. We then applied the Moran process, a stochastic birth-death model, to calculate distributions of accumulation times and to extract key parameters. The Moran model reproduced the distributions obtained from stochastic simulations with high fidelity across different assumptions about mitochondrial regulation. Fitting the model to synthetic data, successfully recovered mutation probability, selection advantage, and the fraction of advantageous mutants. These results establish a methodological framework for quantifying mtDNA mutant dynamics from single-cell transcriptomic data and provide a foundation for analysing large experimental datasets in ageing research.

## Introduction

A hallmark of the ageing process is the accumulation of mitochondrial DNA (mtDNA) deletion mutants in post-mitotic cells such as neurons and skeletal muscle fibres^1–11^. Numerous single-cell and microdissection studies have shown that these deletions, though initially rare, can clonally expand within individual cells to levels well above the threshold required to impair respiratory chain function^12–15^. The resulting cytochrome c oxidase (COX) deficiency and electron transport system (ETS) abnormalities are tightly correlated with structural and functional decline of affected cells. This phenomenon has been described across species, including rodents, rhesus monkeys, and humans, establishing mitochondrial genome instability as a conserved and universal feature of mammalian ageing.

Although the occurrence of deletions is well established, the question of how they accumulate to such high intracellular levels remains unresolved. The selective forces or mechanistic processes that allow mutant genomes to outcompete wild-type mtDNA are still debated^16–21^. Early ideas emphasised the so-called ‘vicious cycle’ hypothesis, where defective mitochondria were proposed to produce excess reactive oxygen species (ROS), thereby inducing further mutations^20,21^. However, this model fails to account for the observation that single cells typically harbour a dominant deletion rather than a mixture of multiple mutants^18^. Later explanations have included the ‘survival of the smallest’ hypothesis^22,23^, positing that shorter genomes replicate faster, and the ‘survival of the slowest’ model, suggesting that dysfunctional mitochondria are spared from degradation due to reduced ROS output^19^. Yet, both models face conceptual and experimental limitations. For example, genome size differences are unlikely to substantially influence replication kinetics^24^, and selective mitophagy mechanisms appear to preferentially eliminate, not protect, dysfunctional mitochondria.

A major advance came from mathematical modelling studies showing that stochastic processes alone, specifically random genetic drift under relaxed mtDNA replication, could in principle account for clonal expansion^16,17^. Neutral drift predicts that, given sufficient time and small effective mtDNA copy numbers within individual cells, some mutations arising early in life can randomly reach high frequencies. Such models reproduce the observed prevalence of COX-deficient cells in aged human muscle and brain. However, a fundamental limitation of the neutral drift hypothesis is that it only provides a good fit for long-lived species such as humans. In short-lived animals like mice or rats, the high mutation rates required to generate the experimentally observed number of COX-deficient fibres would also predict the presence of many different mutant species coexisting within the same cell, a pattern that is not seen experimentally^25^. Instead, affected cells typically harbour a single dominant deletion. This inconsistency highlights the need for additional mechanisms beyond drift to explain clonal expansion in short-lived species^26^.

In recent years, this field has developed considerably, and several important modelling studies have broadened the theoretical landscape of mtDNA dynamics. General stochastic theory has clarified how heteroplasmy variance evolves under turnover and feedback control, thereby highlighting the central importance of stochasticity even when mean copy numbers remain stable^27^. Other studies have extended Moran-type approaches to include mitochondrial network state, fission-fusion dynamics, and selective degradation, showing how organelle dynamics can modulate the pace of drift and selection^28^ and allow for the co-existence of mutant and wild-type mtDNAs^29^. More recently, modelling of single-cell mutational spectra has shown that cryptic point mutations can accumulate approximately neutrally in post-mitotic tissues^30^, while other work has provided evidence that positively selected selfish mtDNA variants in replication-control regions can act as intracellular drivers and amplify linked passenger mutations^31^. In addition, deletion-mutant expansion in skeletal muscle fibres has been explained by spatially structured stochastic drift through the ‘stochastic survival of the densest’ mechanism, in which density asymmetries rather than an intrinsic replicative advantage generate effective expansion^32^. These studies have greatly advanced our understanding of mitochondrial genetics in ageing, but they also address biological questions that are distinct from the one considered here.

To address these contradictions, new mechanistic proposals have been put forward. Kowald and Kirkwood proposed that mtDNA replication in metazoans, which is primed via transcription, creates a regulatory vulnerability^26,33^. They suggest that under normal conditions, transcription is subject to feedback inhibition once sufficient respiratory chain subunits are produced. If a deletion removes the gene(s) involved in this feedback loop, the mutant genome escapes regulation, leading to persistently elevated transcription and replication initiation. Such mutants would thus gain a cis-acting replication advantage independent of genome size or ROS production. Consistent with this idea, surveys of deletion spectra across species have repeatedly shown that clonally expanded deletions almost always encompass the ND4 gene, and often ND5, strongly suggesting that these genes are central to the feedback control system^33^. Computational models incorporating this mechanism reproduce the low heteroplasmy observed in short-lived species, the rapid takeover of mutants once they appear, and the emergence of single dominant deletions per cell, thereby resolving many inconsistencies of previous models.

The accumulation of deletion mutants has important physiological consequences. In skeletal muscle, clonal expansions underlie the segmental cytochrome oxidase-deficient regions known as ragged-red fibres. Studies in humans, rhesus monkeys, and rodents have shown that once mutant loads exceed 80–90%, affected fibres display marked atrophy, splitting, and eventually fibre loss, directly contributing to sarcopenia^2,5,12–15^. In neurons of the substantia nigra, clonal deletions are abundant in aged individuals and Parkinson's disease patients, with deletion burdens exceeding 60% tightly associated with COX deficiency^1,3,6^. These findings highlight not only the causal role of mtDNA deletions in age-related tissue decline but also their potential contribution to neurodegeneration.

The temporal dynamics of clonal expansion are therefore of central interest. Does the accumulation of mutants proceed slowly and stochastically throughout life, or do specific mechanisms trigger a rapid takeover once deletions arise in vulnerable genomic regions? Understanding these dynamics is critical, as they determine how long cells can tolerate mutant genomes before crossing the pathogenic threshold. The transcription-coupled replication model predicts a biphasic process: an initial lag until a deletion removing the feedback gene arises, followed by a rapid exponential expansion phase. This would result in relatively similar accumulation times across mammalian species despite differences in lifespan^33^.

In this study, we propose a novel approach to infer accumulation times of mtDNA deletion mutants from cross-sectional single-cell RNA sequencing (scRNAseq) data. Since the present study is concerned with deletions at the DNA level, data from single-cell DNA sequencing techniques would be most appropriate for the analysis. In recent years, such techniques have been described in the literature^34^, including approaches that concentrate specifically on mtDNA^35,36^. Indeed, recent work has applied single-cell mtDNA sequencing to hepatocytes from young and old mice, thereby demonstrating the feasibility of studying age-related mtDNA mutational patterns at single-cell resolution^31^. However, for the type of parameter inference pursued here, such data are still not fully sufficient, because our parameter fitting approach requires cross-sectional measurements at multiple ages rather than comparison of only two age groups. Since deletions in the mitochondrial genome directly lead to deletions in the corresponding transcripts, patterns in mitochondrial gene expression provide an indirect but informative readout of the underlying mutational landscape. By analysing these patterns across large numbers of cells, it becomes possible to extract quantitative information about mutant accumulation dynamics that cannot be accessed by longitudinal measurements.

This paper is organised as follows. In the first section, ‘*Obtaining longitudinal accumulation dynamics from cross-sectional scRNAseq data’*, we introduce the conceptual framework that links single-cell transcriptomic measurements to underlying mutant accumulation trajectories and illustrate how cross-sectional snapshots can encode information about longitudinal dynamics. In the next section, ‘*Generation of synthetic data’*, we describe two stochastic models of the mitochondrial life cycle that incorporate replication, mutation, and degradation, and use these models to generate synthetic datasets that serve as gold standards for method validation. The advantage of using synthetic data is that the true parameters underlying the system are known, allowing us to rigorously test the accuracy of inference methods. We then analyse an additional key observable in the section ‘*Distribution of unique mutants’*, where we investigate how many distinct deletion species are expected to coexist within individual cells. Using the synthetic data, we show that the number of unique mutant types follows a Poisson distribution and examine how its single parameter depends on mutation rate, selection advantage, and age. In the subsequent section, ‘*Using the Moran process to model accumulation times’*, we demonstrate that a reduced stochastic framework based on the Moran process^37–39^ accurately reproduces the accumulation time distributions obtained from the more detailed simulations. Finally, in ‘*Fitting the Moran process to real world data’*, we show how the Moran model can be fitted to cross-sectional summary statistics, enabling the extraction of mutation probabilities, selection advantages, and associated confidence intervals in a way that is directly applicable to experimental scRNAseq data.

Together, these results establish a coherent pipeline that links single-cell transcriptomic measurements to quantitative estimates of mtDNA mutant dynamics, providing the foundation for the experimental application presented in a forthcoming companion study. In contrast to recent mechanistic studies that focus primarily on the origin of drift, selection, network-state effects, or spatial propagation, the present manuscript is concerned with the inference of mutant accumulation times and associated parameters from cross-sectional single-cell data.

## Results

### Obtaining longitudinal accumulation dynamics from cross-sectional scRNAseq data

Measuring the amount of mitochondrial DNA (mtDNA) mutants inside a single cell inevitably destroys that cell, making it impossible to obtain accumulation times by repeatedly following the same cell over time. This raises the critical methodological question of whether it is possible to derive such information from cross-sectional data, such as single-cell RNA sequencing (scRNAseq). Ideally, one would like to obtain direct quantitative measurements of mutant and wild-type genomes at the DNA level. Single-cell DNA sequencing (scDNAseq) would in principle provide the necessary resolution, and indeed, numerous areas of biology, from cancer genomics to developmental lineage tracing, would benefit from such approaches^40^. However, technical obstacles have historically limited the reliability of scDNAseq. Problems such as incomplete genome coverage, amplification bias, allelic dropout, and high error rates have restricted its widespread use, especially in applications where quantitative accuracy is essential. Recent methodological innovations have begun to address these challenges, for instance, through intracellular genomic amplification strategies that amplify DNA within intact, permeabilized cells, thereby avoiding some of the bottlenecks of traditional single-cell DNA protocols^41^. Despite these promising advances, scDNAseq is still not yet available at the scale and accuracy required to systematically investigate mtDNA deletions across large numbers of individual cells.

Given these limitations, single-cell transcriptomics provides a practical and informative alternative. The rationale is that deletions at the DNA level are faithfully reflected at the RNA level. This creates a direct link between DNA-level deletions and their transcriptional consequences. Thus, the relative abundance of mutant versus wild-type mtDNA can be approximated by measuring the ratio of transcripts that contain a deletion versus those that don’t. Because the selective advantage considered here is itself linked to altered transcriptional regulation, this approximation is not expected to be exact. In particular, selectively advantaged mutants may be somewhat overrepresented at the RNA level. However, as shown in the Methods, the relationship between RNA-based and DNA-based heteroplasmy can be quantified analytically and remains sufficiently well behaved for scRNAseq to provide a useful first approximation. While not a perfect proxy, since RNA stability and processing may introduce additional layers of complexity, this approach nonetheless offers a unique window into the intracellular dynamics of mtDNA populations. Importantly, scRNAseq can be applied at high throughput, providing data from tens of thousands of cells in parallel, which is essential to capture the rare and stochastic events underlying clonal expansion of mtDNA deletions.

**Fig. 1:**
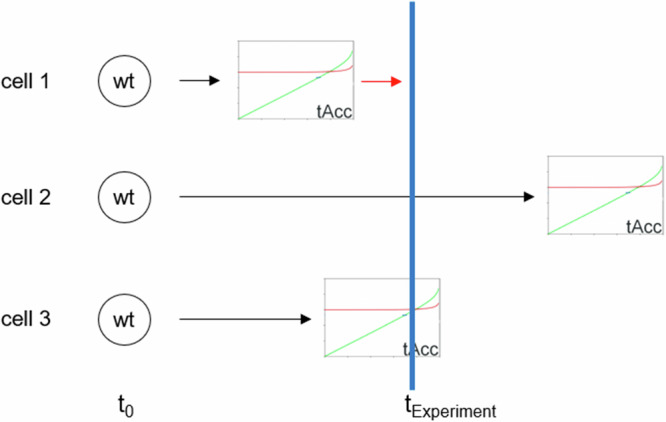
Individual cells start with only wild type mtDNA, but there is a certain probability per replication, *mutProb*, that a mutation occurs. From that moment on mutants (green) accumulate until the cell is taken over after tAcc days. tAcc can be a fixed number or come from a distribution. At time tExp (blue bar), a single cell NGS experiment is performed, which in this case would reveal that cell1 is completely taken over, cell2 is still wild-type and cell3 is caught in the middle of mutant accumulation.

Figure 1 illustrates the conceptual framework using three example cells, plotted with time on the horizontal axis. Initially, all cells contain exclusively wild-type mtDNA molecules. At each round of replication, however, there is a finite probability that a deletion mutation occurs. This generates the first mutant molecule, which can then expand within the cell population of mtDNAs. Over time, the mutant genomes may clonally dominate, progressively displacing the wild type. This process is symbolized in the inset diagram, with wild-type genomes shown in red and mutant genomes in green, loosely following simulation results from ref. ^26^. If a scRNAseq experiment is performed at a given time point (marked by the vertical blue bar), the mtDNA composition of each cell is captured indirectly through its transcriptome. In the example shown, cell 1 has undergone complete takeover by mutant genomes and therefore produces only mutant-associated transcripts. Cell 2 has not yet experienced a mutation event and thus shows only wild-type signals. Cell 3 represents an intermediate state, with transcripts derived from both wild-type and mutant genomes, reflecting a cell in the midst of the accumulation process.

This schematic makes clear that the probability of observing cells at different stages of mutant takeover depends on a set of underlying parameters, such as the mtDNA half-life, the mutation rate, and any selective advantage carried by the deletion mutants. Crucially, this also implies that, given sufficient single-cell transcriptomic data, one can invert the problem and deduce the relevant parameters from the distribution of mutant-to-wild-type ratios observed across many cells. In this way, scRNAseq data provide not only a snapshot of the heterogeneity of mtDNA populations across individual cells but also a means to extract quantitative information about the fundamental processes governing the accumulation of mitochondrial mutants.

### Generation of synthetic data

To test whether accumulation times of mtDNA mutants can indeed be inferred from cross-sectional data, we simulated the mitochondrial life cycle, comprising replication, mutation, and degradation, using two distinct stochastic models. This strategy provides access to longitudinal trajectories of mutant expansion, which are experimentally inaccessible. From the simulated data, we obtained distributions of mutant accumulation times that serve as a gold standard against which our analytical approach can be evaluated.

Model-1, originally developed in ref. ^26^, consists of three ordinary differential equations (ODEs) describing the time evolution of wild-type (wt) and mutant (mt) mtDNAs as well as ATP. In this formulation, the cell strives to maintain a fixed ATP level, which results in an expanding mtDNA population size as mutant genomes accumulate. Model-2 represents a streamlined variant of Model-1 and involves only two equations, one each for wild-type and mutant mtDNAs. Here the regulation is imposed at the level of total mtDNA copy number, i.e. the cell maintains a constant population size, N, throughout the accumulation process, independent of ATP dynamics.

In both models mutant accumulation arises from the assumption that mutant genomes replicate at a higher rate than wild-type (*selAdv* > 0). A detailed discussion of the biological justification for these assumptions, as well as the parameter values used (e.g. *N* = 1000, mtDNA half-life = 10 days), is provided elsewhere^26,33^. Although the ODE formulation is mathematically concise, we implemented the models as stochastic simulations in Java to capture random mutation events and probabilistic replication and degradation. The simulation code is available from our GitLab repository. This stochastic framework also allows us to account for different classes of deletion mutants. Based on our earlier predictions, only those deletions that disrupt genes involved in the proposed transcriptional feedback mechanism confer a selective replication advantage, while deletions in other regions provide no advantage and can only persist by neutral drift^26^. Consequently, the programme explicitly tracks wild-type genomes, mutants with a replication advantage, and mutants without advantage.

**Model-1**

1 $$\frac{{dwt}}{{dt}}=\frac{{wt}(t)}{1+{ATP}(t)/c}-\frac{\mathrm{ln}2}{{halfL}}\cdot {wt}(t)$$

2 $$\frac{{dmt}}{{dt}}=\frac{(1+{selAdv})\cdot {mt}(t)}{1+{ATP}(t)/c}-\frac{\mathrm{ln}2}{{halfL}}\cdot {mt}(t)$$

3 $$\frac{{dATP}}{{dt}}=f\cdot {wt}(t)-{v}_{2}\left({wt}(t)+{mt}(t)\right)-{v}_{1}\cdot {ATP}(t)$$

**Model-2**

4 $$\frac{{dwt}}{{dt}}=\frac{{wt}(t)}{(1+{selAdv})\cdot {mt}(t)+{wt}(t)}\cdot (N-{mt}\left(t\right)-{wt}(t))-\frac{\mathrm{ln}2}{{halfL}}\cdot {wt}(t)$$

5 $$\frac{{dmt}}{{dt}}=\frac{(1+{selAdv})\cdot {mt}(t)}{(1+{selAdv})\cdot {mt}(t)+{wt}(t)}\cdot (N-{mt}\left(t\right)-{wt}(t))-\frac{\mathrm{ln}2}{{halfL}}\cdot {mt}(t)$$

Employing two models with distinct regulatory assumptions allowed us to assess the robustness of our analytical method. Since the true mechanisms controlling mitochondrial biogenesis and turnover remain incompletely understood, it is important to verify that the inference procedure is not overly sensitive to model-specific details. For both models, we simulated the fate of 100000 cells over a 3-year period, approximating the lifespan of a mouse, and recorded the time from the first appearance of a mutant until takeover by mutants. Simulations varied the selection advantage (*selAdv*), the mutation probability per replication (*mutProb*), and the fraction of mutations generating an advantageous mutant (*fracAdv*). Takeover was defined as the point at which mutants reached 90% abundance; in Model-1, this coincided with ATP collapse (ATP = 0), while in Model-2 it was explicitly imposed as the 90% threshold.

**Fig. 2:**
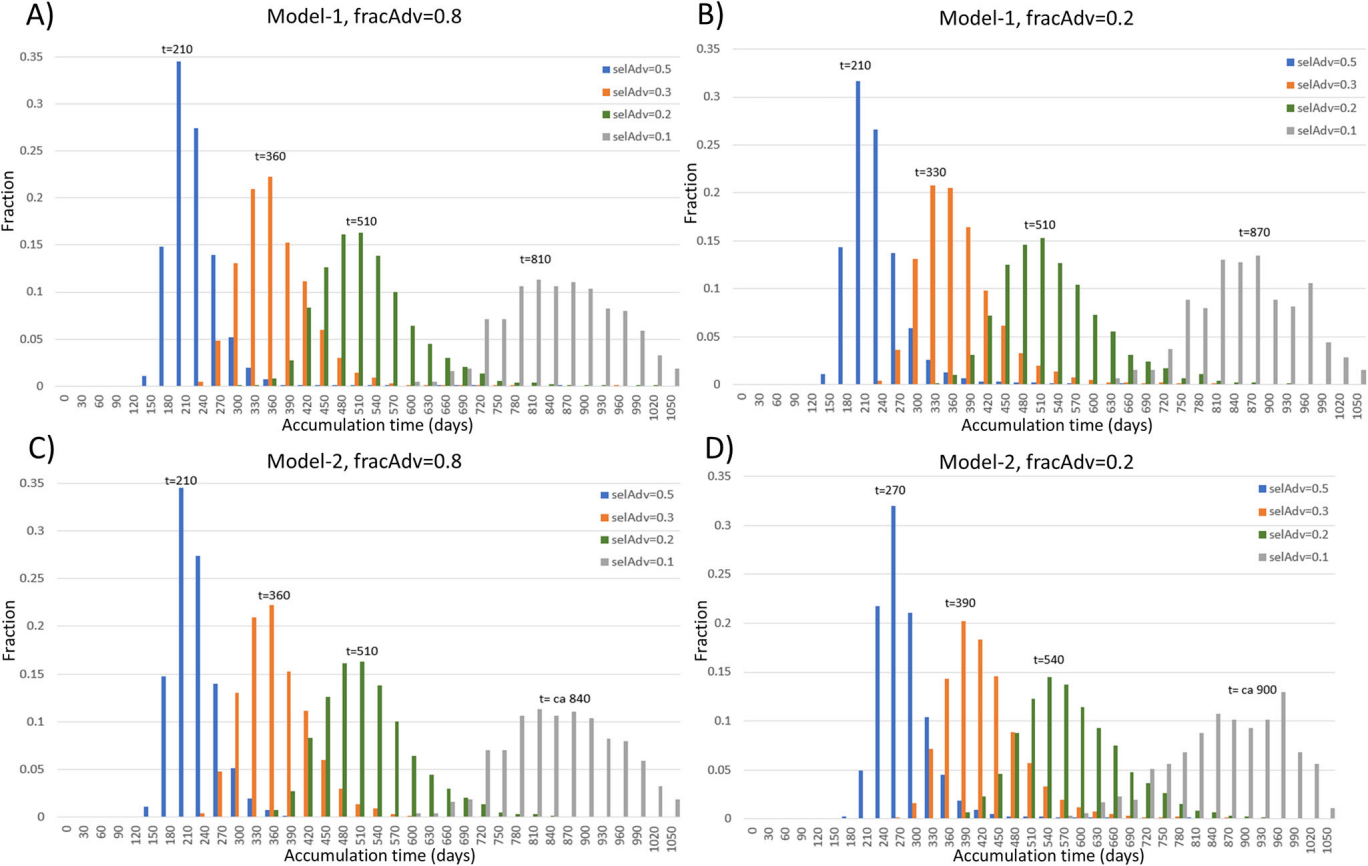
Distributions of accumulation times for mitochondrial deletion mutants (from first occurrence until 90% prevalence) obtained from stochastic simulations. **A**–**D** show results from two different simulation models of the mitochondrial life cycle (see main text). Each histogram represents the distribution of accumulation times. **A**, **C** Depict simulations where 80% of mutations confer a selective advantage, while **B**, **D** show simulations where only 20% of mutations produce advantageous mutants.

Figure 2 summarises the resulting distributions of accumulation times for different parameter settings. Please note that these times start from the occurrence of the first mutant mtDNA molecule and not from the birth of the animal. Histograms are binned in 30-day intervals, corresponding to the snapshot frequency of the simulation. For cases with *fracAdv* = 0.2 (Fig. 2B, D), we increased the mutation rate from 6.8e-6 to 3e-5 per replication to ensure sufficient numbers of takeover events among the 100000 simulated cells; otherwise, the number of affected cells would have been too low to yield reliable histograms.

Overall, the results show that accumulation times are approximately normally distributed with a long right-hand tail. As expected, higher selection advantages shortened the takeover time. Notably, the two models produced very similar distributions (compare Fig. 2A, B with Fig. 2C, D), suggesting that the precise details of mitochondrial regulation, whether based on ATP homoeostasis or fixed copy number, are not critical determinants of mutant accumulation kinetics. Likewise, varying the fraction of mutants with selective advantage (*fracAdv*) had little impact on the distributions. In fact, inspection of the simulation data revealed that none of the takeover events was caused by mutants without selective advantage; these genomes never succeeded in displacing wild-type mtDNAs. This outcome is consistent with our earlier finding that accumulation via drift alone is not feasible in short-lived species such as mice or rats^25^.

### Distribution of unique mutants

Using the synthetic datasets, we can not only extract accumulation times for deletion mutants, as shown in the previous section, but also characterise an equally important feature of mitochondrial mutational dynamics: the distribution of unique mutant species present in individual cells at old age. As noted earlier, post-mitotic cells typically harbour only one, or at most a very small number, of different deletion types. This empirical observation is a major constraint on any mechanistic hypothesis seeking to explain mtDNA mutant accumulation, because several proposed models, most notably neutral drift, predict a much larger diversity of mutants within single cells, especially in short-lived species^25,26^. Understanding why only a few deletion species dominate is therefore essential for distinguishing between competing hypotheses.

**Fig. 3:**
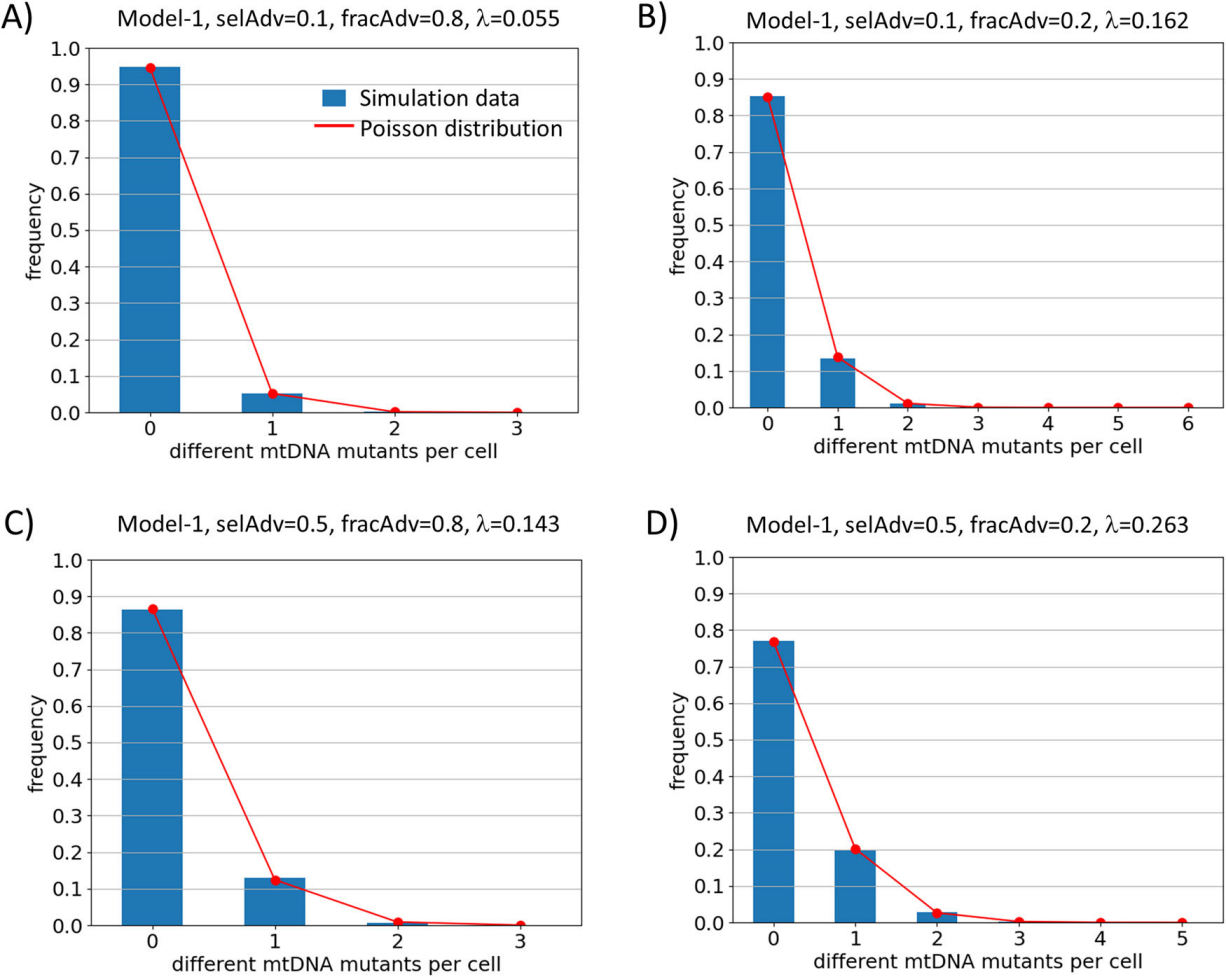
Distributions of the number of unique deletion mutants per cell at the end of a three-year mouse lifespan, obtained from stochastic simulations of Model-1. A value of zero indicates cells containing only wild-type mtDNAs. Panels **A**–**D** show results for all four possible combinations of low and high values of selection advantage (*selAdv*) and fraction of advantageous mutants (*fracAdv*). Blue bars show the simulation results, while red lines indicate the corresponding Poisson distributions calculated using the mean number of unique mutants observed in each simulation.

To explore this, we analysed the state of each simulated cell at the end of a 3-year simulation period (corresponding to the lifespan of a mouse) across a range of parameter combinations for selection advantage (*selAdv*) and the fraction of mutations that lead to advantageous mutants (*fracAdv*). For each parameter set, we counted how many distinct mutant types were present in every cell. The resulting distributions, shown in Fig. 3, reproduce earlier observations^26^, i.e. whenever deletion mutants possess a selection advantage, the overwhelming majority of cells contain only a very small number of unique deletion species.

Because the appearance of deletion mutants is driven by rare mutation events occurring during replication, we next asked whether the number of unique deletion types per cell might follow a Poisson distribution. The Poisson process is the canonical model for rare, independent events occurring at a constant rate, and it is therefore a natural candidate for describing the stochastic generation of deletion species. To test this idea, we computed the mean number of unique mutants across the 100,000 simulated cells for each parameter condition, and used this mean as the Poisson rate parameter (λ). We then overlaid the resulting Poisson probability mass function (in red) onto the histogram of observed values (blue bars) in Fig. 3. The agreement is remarkably close across all parameter sets examined. At first glance, it may appear intuitive that rare mutation events would produce a Poisson-distributed number of unique deletion types. However, the excellent agreement observed here is far from obvious. The mitochondrial life cycle is not a simple accumulation of independent mutation events, instead it includes several nonlinear processes that could in principle distort or completely break the Poisson pattern. First, once a deletion mutant arises, it undergoes replication, competition, and degradation within the mitochondrial population. Mutants with a selective advantage tend to expand rapidly, effectively suppressing the fixation of other mutants arising later. This introduces interactions between mutant lineages, violating the independence assumption of a pure Poisson process. Second, degradation (mitophagy) and the feedback-regulated replication dynamics of wild-type and mutant genomes further influence the fate of both mutant classes and their coexistence. These processes introduce time-dependent biases, making it unclear whether the number of unique mutants should retain a simple distributional form. Third, the presence of two classes of mutants, with and without selection advantage, adds additional structure, as the former tend to dominate the population while the latter persist only transiently.

**Fig. 4:**
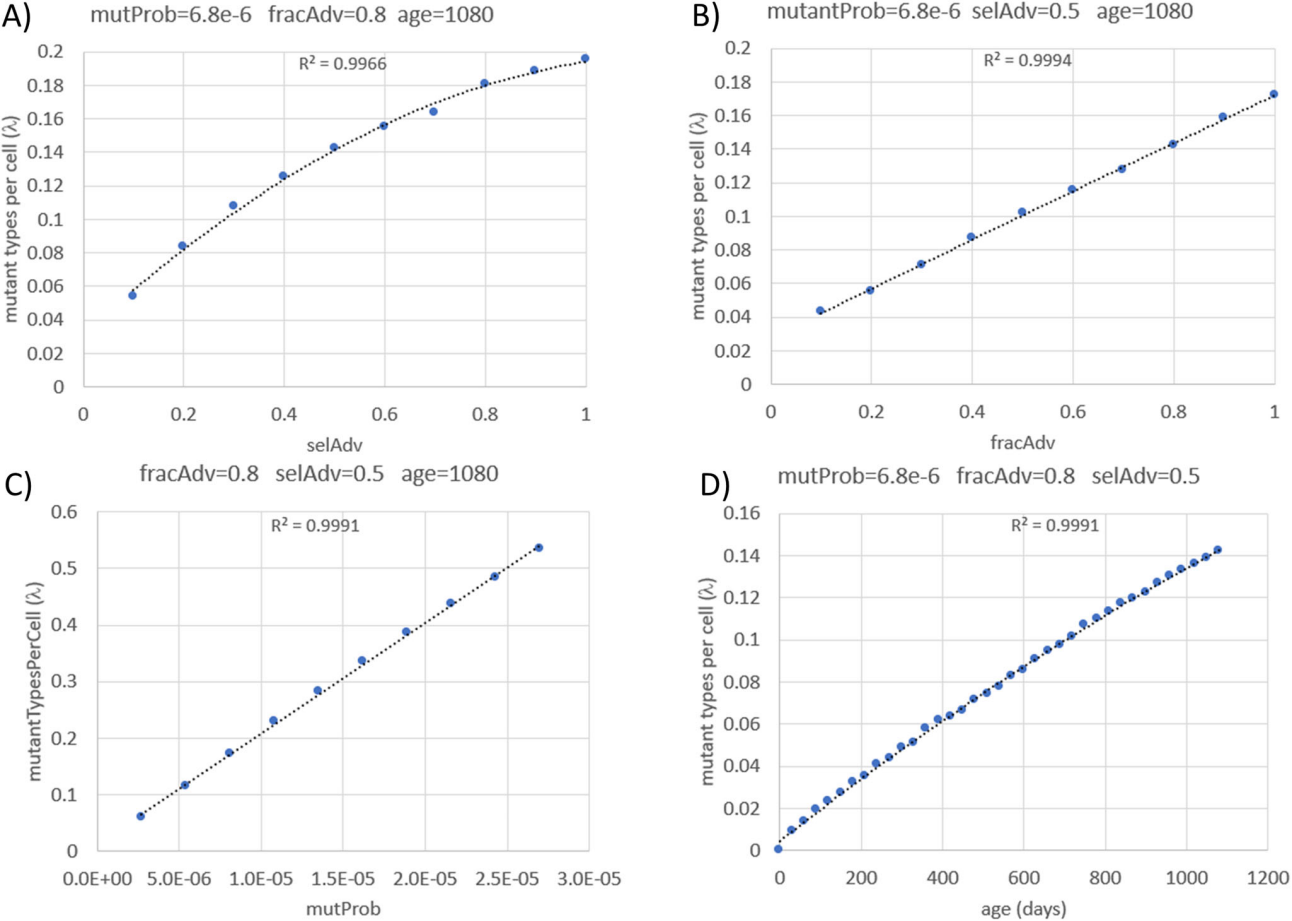
Dependence of the mean number of unique deletion mutants (λ) on key model parameters. For each panel, *λ* was calculated from the synthetic data as the average number of distinct deletion species per cell at the end of the simulation period. Linear fits highlight the nearly perfect proportional dependence of *λ* on fracAdv (**B**) and mutProb (**C**), whereas quadratic trendlines capture the nonlinear relationships with selAdv (**A**) and age (**D**).

A direct consequence of the finding that the distribution of unique mutant types follows a Poisson distribution is that it can be fully characterised by a single parameter, λ, representing the mean number of distinct deletion species present per cell. This greatly simplifies the description of mutational diversity and reduces the problem to understanding how λ depends on the underlying biological parameters of the system. To investigate this, we systematically varied the key model parameter, selection advantage (*selAdv*), fraction of advantageous mutations (*fracAdv*), mutation probability (*mutProb*), and age, and computed λ for each condition based on the synthetic data. The resulting relationships are shown in Fig. 4.

The mean number of unique mutants *λ* increases almost perfectly linearly with both the fraction of advantageous mutants (*fracAdv*) and the mutation probability (*mutProb*). In contrast, λ exhibits a nonlinear, nearly quadratic dependence on both selection advantage (*selAdv*) and age. These relationships suggest that the number of unique deletion species in a cell results from a balance between stochastic mutational input (linear effects) and the amplification dynamics driven by selection and time (non-linear effects). The observation that such clear mathematical patterns emerge from a complex biological model underscores the robustness of the underlying processes and suggests that similar scaling laws may hold in vivo.

### Using the Moran process to model accumulation times

The synthetic data generated in the previous section provide a test-case for real scRNAseq measurements, allowing us to test whether it is possible to recover key parameters of the model. Because the true parameter values are known in this case, the synthetic data serve as a gold standard for evaluating the accuracy of our inference approach.

To perform this inference, we used the Moran process^37–39^, a classical stochastic model from population genetics that describes how a new variant spreads in a finite population of constant size. The mathematical details of the model, including the derivation of accumulation time distributions using recursive first-step analysis, are provided in the Methods section. For comparison with the synthetic data we computed the distribution of accumulation times under the Moran process. Instead of requiring full fixation, we stopped the calculation once mutants reached 90% of the population, which corresponds to our operational definition of takeover in the simulations.

**Fig. 5:**
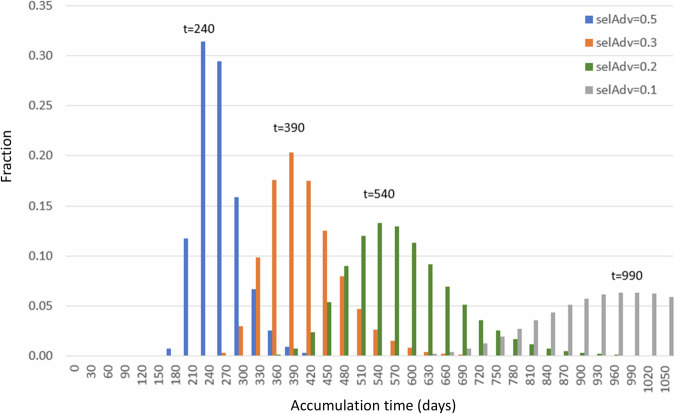
Distributions of accumulation times for mitochondrial deletion mutants, defined as the time interval from first occurrence of a mutant until it reaches 90% abundance, as predicted by the Moran model. Each histogram shows the probability distribution of accumulation times for a given value of the selection advantage *selAdv*, with all other parameters kept constant. The distributions are broad and right-skewed, and increasing selection advantage shifts the peak toward shorter accumulation times, indicating faster takeover dynamics.

The distributions derived from the Moran model (Fig. 5) closely match those obtained from the stochastic simulation models (Fig. 2). Only in the case of a very small selection advantage (*selAdv* = 0.1) do noticeable discrepancies appear, i.e. the synthetic data show a peak probability shifted toward earlier times. This difference is not due to a failure of the Moran model but rather to an observational bias in the simulations. Because the simulations were limited to three years, the approximate lifespan of a mouse, extremely long takeover times could not be captured. Truncating the distribution in this way artificially inflates the probabilities of earlier takeover (we verified this by performing simulations for 6 years, which removed the discrepancies). In contrast, the Moran-based calculation was extended until the entire distribution was observed.

These results demonstrate that the Moran process can successfully reproduce the accumulation time distributions generated by two different, more detailed, stochastic simulations. Despite the fact that the true regulation of mitochondrial biogenesis and turnover is not fully understood, this agreement suggests that the Moran model provides a robust and tractable framework for analysing the accumulation dynamics of mitochondrial mutants. Importantly, while synthetic data allows direct measurement of accumulation times by following individual simulated cells, this is not possible experimentally because cells are destroyed during scRNAseq measurement. In the next section, we therefore show how the Moran model can be exploited to infer the selective advantage of mtDNA mutants from cross-sectional data, thereby bridging the gap between theory and experiment.

### Fitting the Moran process to real world data

**Fig. 6:**
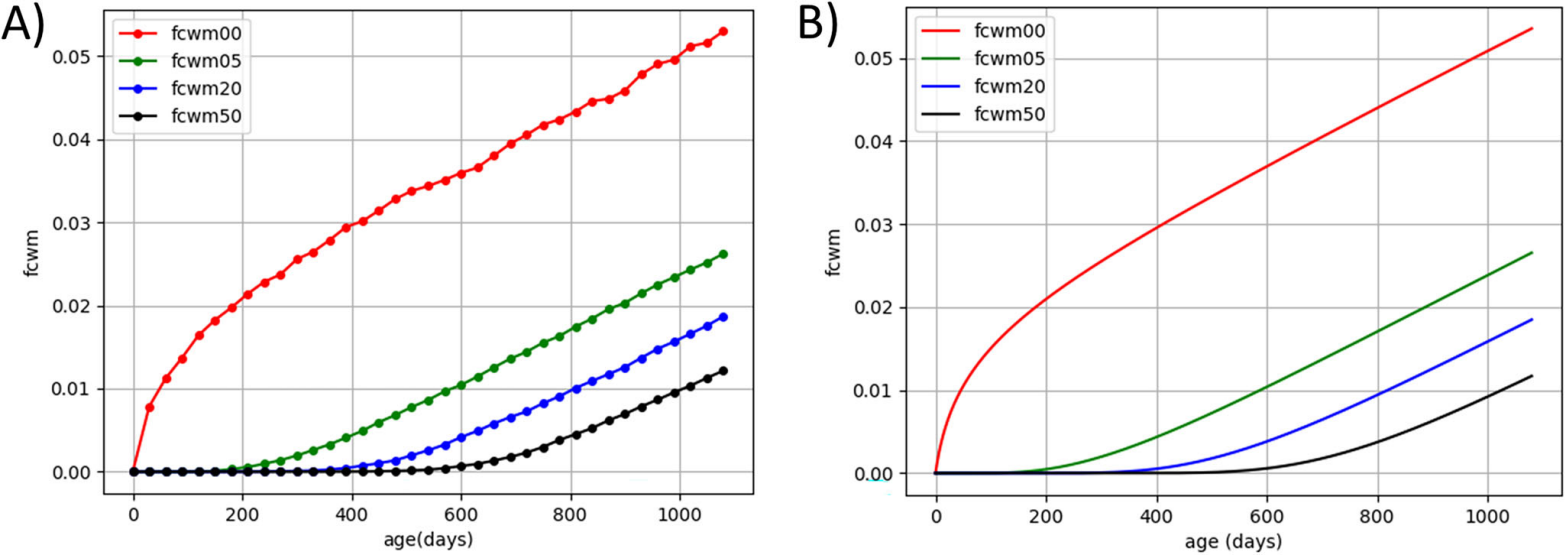
Comparison of mutant acccumulation in stochastic simulations vs Moran process. **A** Fraction of cells with more than 0%, 5%, 20% and 50% of mutant mtDNAs as function of time for Model-1 using the parameter values *selAdv* = 0.1, *mutProb* = 6.8e-6 and *fracAdv* = 0.8. Data values are exported and displayed in 30 day intervals. **B** The same type of information generated from the forward-master equation of the Moran process.

Longitudinal single-cell trajectories, such as those obtained in the previous section from simulations, are not available in experimental scRNAseq data. Instead, we only have cross-sectional information so that we can measure the fraction of cells that harbour different proportions of deletion mutants (or, equivalently, transcripts with deletions) only at specific time points. This information can also be derived from our synthetic data as well as the Moran process (see Fig. 6). To demonstrate this, we utilised the forward master equation of the Moran process to compute the probability distribution over mutant counts at any time *t*. As detailed in the Methods section, this approach captures continuous turnover and competition among genome classes, mapped to physical time units. From this, summary measures can be extracted, such as the fraction of cells with more than a given percentage of mutants.

Figure 6B shows that when the true parameter values are used, the Moran process reproduces the synthetic data curves from Fig. 6A with high accuracy. For real experimental data, however, the parameter values are not known a priori and must instead be inferred by fitting the model to the observed data. We therefore performed parameter estimation by fitting three key parameters, mutation probability (*mutProb*), selection advantage (*selAdv*), and the fraction of advantageous mutants (*fracAdv*), to the data. This was achieved by minimising the sum of squared differences between the Moran predictions and the empirical curves using a differential evolution algorithm (see ‘Methods’).

Table 1 summarises the ground truth parameter values together with the estimated mean, coefficient of variation (CV), and 95% confidence intervals obtained via parametric bootstrapping. For all three parameters, the estimated means are very close to the true values used to generate the data, indicating that the fitting procedure is unbiased in this parameter regime. The CV values are small, in particular for the mutation probability *mutProb* and the selection advantage *selAdv*, demonstrating high statistical precision.

**Table 1:** Parameter estimates obtained via parametric bootstrapping (*N* = 40) of 100,000 simulated cells

Name	Ground Truth	Estimate Mean	Estimate CV (%)	95% CI
*mutProb*	6.8e-6	6.74e-6	1.71	[6.55e-6, 6.99e-6]
*selAdv*	0.1	0.108	4.51	[0.094, 0.117]
*fracAdv*	0.8	0.87	7.41	[0.71, 1]

The fraction of advantageous mutations (*fracAdv*) shows a larger CV and an asymmetric confidence interval, with the upper bound reaching the imposed maximum value of 1. This behaviour indicates partial non-identifiability of *fracAdv* near its upper boundary. Once the fitting procedure reached a large value for *fracAdv*, the data contain limited information to distinguish, for example, *fracAdv* = 0.9 from *fracAdv* = 1. Importantly, this does not reflect a failure of the fitting procedure, but rather a structural limitation of the available observables under this parameter regime.

**Fig. 7:**
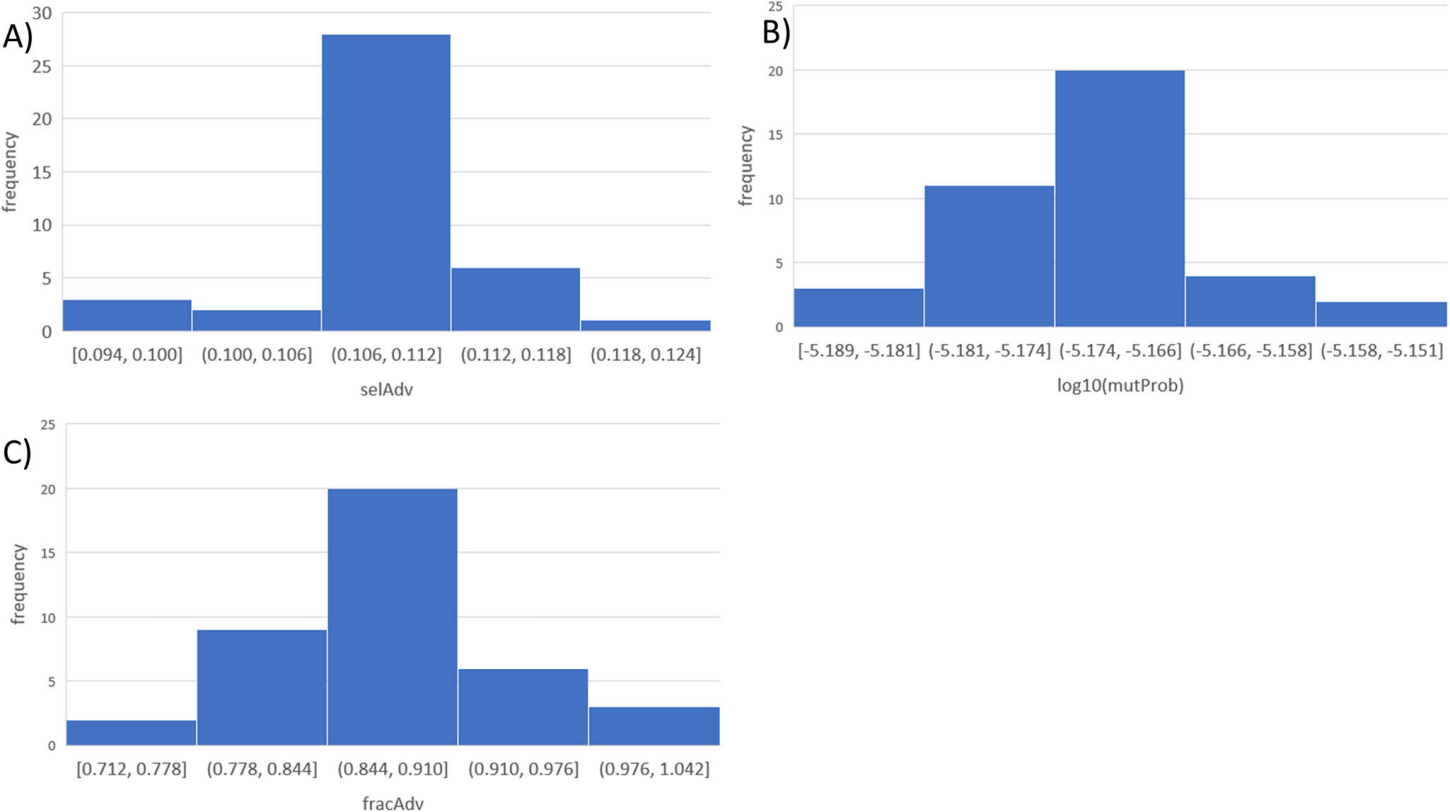
Distribution of parameter estimates. Histograms show the estimated values of (**A**) selection advantage (*selAdv*), (**B**) mutation probability (*mutProb*), and (**C**) fraction of advantageous mutations (*fracAdv*) obtained from 40 independent bootstrap data sets, each based on 100,000 simulated cells. The mutation probability is displayed on a logarithmic scale. All distributions are unimodal and centred close to the corresponding ground truth values, indicating unbiased estimation and stable convergence of the fitting procedure.

Figure 7 shows histograms of the estimated parameter values obtained from 40 bootstrap data sets (with *mutProb* displayed on a logarithmic scale). All three distributions are unimodal and approximately symmetric around a single maximum, with no evidence for secondary optima or multimodality. This indicates that the optimisation landscape is well behaved and that the stochastic optimisation procedure converges consistently to the same region of parameter space. The relatively narrow spread of the distributions further confirms the high precision of the estimates for data sets of this size.

In a forthcoming publication^42^, we apply the same fitting framework to experimental single-cell transcriptomics data, which are typically based on substantially smaller numbers of cells, often in the range of a few thousand. To assess how parameter uncertainty scales with reduced sample size, and to obtain confidence interval estimates that are relevant for such experimental settings, we repeated the parametric bootstrapping procedure using synthetic data sets based on only 10,000 simulated cells.

The results are summarised in Table 2. As expected, the estimated means remain close to the ground truth values, demonstrating that the fitting procedure remains unbiased even at lower cell numbers. However, the CV values increase and the confidence intervals widen for all three parameters, reflecting the higher stochastic variability inherent to smaller sample sizes. The key idea underlying this analysis is that, when fitting real experimental data with a comparable number of cells, the confidence intervals obtained from synthetic data of the same size provide a quantitative estimate of the expected statistical uncertainty. Since the synthetic data are generated from the same stochastic model that underlies the inference procedure, these confidence intervals can be interpreted as model-conditional minimal uncertainty estimates for the experimental parameter fits. Of course, biological and experimental variability can increase these estimates further.

**Table 2:** Parameter estimates obtained via parametric bootstrapping (*N* = 40) of 10000 simulated cells

Name	Ground Truth	Estimate Mean	Estimate CV (%)	95% CI
*mutProb*	6.8e-6	6.65e-6	4.24	[6.11e-6, 7.22e-6]
*selAdv*	0.1	0.107	9.74	[0.089, 0.125]
*fracAdv*	0.8	0.87	13.03	[0.65, 1]

Finally, to demonstrate the robustness of the fitting approach across different regions of parameter space, we repeated the entire procedure (again based on 10,000 simulated cells) using a second set of parameter values that differs substantially from the first (higher mutation probability, stronger selection, and a lower fraction of advantageous mutations). The results are shown in Table 3. As in the previous analyses, the estimated means agree well with the corresponding ground truth values, indicating that the fitting procedure generalises well across parameter regimes. Notably, the confidence interval for *fracAdv* is considerably narrower in this case. This can be explained by the fact that *fracAdv* = 0.2 lies well within the interior of the admissible parameter range, far from the upper boundary at 1. Consequently, the data contain sufficient information to constrain *fracAdv* more tightly, and the identifiability issues observed in the high-*fracAdv* regime are no longer present. Another noteworthy observation is that the upper confidence bound for *mutProb* lies slightly below the true value. This behaviour is consistent with finite-sample variability in the bootstrap procedure and reflects a small downward bias of the estimator under this specific parameter combination. Importantly, the true value still lies close to the confidence interval, and repeated bootstrap analyses with different random seeds yield similar results, indicating that this effect is modest and does not compromise the overall robustness of the inference.

**Table 3:** Parameter estimates obtained via parametric bootstrapping (*N* = 40) of 10000 simulated cells for a 2nd set of parameter values

Name	Ground Truth	Estimate Mean	Estimate CV (%)	95% CI
*mutProb*	3e-5	2.9e-5	1.78	[2.80e-5, 2.99e-5]
*selAdv*	0.5	0.538	8.05	[0.461, 0.628]
*fracAdv*	0.2	0.21	8.14	[0.18, 0.24]

In summary, these results demonstrate that by fitting the Moran model to cross-sectional data, as would be available from scRNAseq experiments, it is possible to recover parameters that provide important insights into the occurrence and accumulation of mtDNA deletion mutants. The estimates of selection advantage in particular provide direct insight into whether mutant accumulation is driven by random drift or positive selection (which allows to differentiate between different hypotheses), and they can be used to calculate the expected distribution of accumulation times from the first appearance of a mutant until its eventual takeover of the mitochondrial population.

## Discussion

The accumulation of mitochondrial DNA (mtDNA) deletion mutants in post-mitotic cells has long been recognized as a hallmark of ageing^1–11^. Numerous studies in skeletal muscle, neurons, and other tissues across mammals have shown that mtDNA deletions, although rare in early life, can clonally expand within single cells to levels that surpass the threshold required for respiratory chain dysfunction. Once mutant loads exceed approximately 80–90%, oxidative phosphorylation collapses, leading to cytochrome c oxidase (COX) deficiency, atrophy, and ultimately the loss of affected cells. These events are central to age-related sarcopenia and neurodegeneration. Yet, despite decades of research and substantial recent progress in modelling mitochondrial population dynamics, the fundamental question of why certain deletion mutants are able to outcompete wild-type genomes and accumulate to such high levels has remained unresolved.

Several mechanistic hypotheses have been advanced. Early ‘vicious cycle’ models proposed that respiratory dysfunction increased reactive oxygen species (ROS) production, thereby generating further mtDNA damage^20,21^. While appealing, this idea is inconsistent with the observation that single cells typically harbour a dominant deletion species rather than a heterogeneous mixture of multiple mutants. Other models emphasised structural or functional advantages of mutants. The ‘survival of the smallest’ hypothesis suggested that shorter genomes replicate more rapidly, whereas the ‘survival of the slowest’ model argued that dysfunctional mitochondria escape turnover by producing fewer damaging ROS. However, both models face serious conceptual and empirical limitations. Genome size differences appear too small to meaningfully affect replication kinetics, and mitophagy mechanisms generally target rather than protect dysfunctional organelles.

Neutral drift offered a more parsimonious explanation. Mathematical models demonstrated that random replication and degradation under relaxed control could, in principle, generate clonal expansions of mtDNA mutations^16,17^. Drift is particularly effective when copy numbers are low and sufficient time is available for stochastic fluctuations to carry a mutant to dominance. These models successfully reproduced the frequency of COX-deficient cells in long-lived species such as humans. However, they fail for short-lived species like mice or rats, where drift would predict multiple mutant species per cell, a pattern not observed experimentally. This discrepancy highlighted the need for an additional selective mechanism.

To address this gap, a transcription-coupled replication model was proposed^26,33^. In mammalian mitochondria, replication is primed by transcripts, and transcription is proposed to be subject to negative feedback once sufficient respiratory chain subunits are produced. Deletions that remove the genes responsible for this feedback loop escape regulation, leading to persistently high transcription and replication initiation. Mutants encompassing the ND4 or ND5 genes, repeatedly identified in expanded deletions across species, are therefore predicted to gain a cis-acting replication advantage. Computational models incorporating this mechanism explain both the low heteroplasmy levels observed in short-lived animals and the dominance of single deletions per cell, while still remaining consistent with patterns in long-lived species.

The key prediction of this framework is that once such a mutant arises, its accumulation time from first occurrence until cellular takeover should be relatively short, on the order of several months, and similar across species. Knowing this accumulation time is therefore crucial for distinguishing between competing hypotheses of mutant expansion. Unfortunately, the accumulation time cannot be measured directly, since experimental approaches to quantify mtDNA mutants destroy the cell. Longitudinal measurements on the same cell are therefore impossible. In this study, we addressed this challenge by proposing that accumulation times can be inferred indirectly from cross-sectional measurements such as single-cell RNA sequencing. With sufficient sampling across many cells, it should be possible to reconstruct the distribution of accumulation states and, from this, infer the parameters governing the expansion process. A limitation of the present implementation is that heteroplasmy is inferred from RNA rather than measured directly at the DNA level. Under the transcription-coupled replication hypothesis, this introduces a systematic but bounded bias, because mutants that escape feedback inhibition are expected to be somewhat overrepresented in the transcript pool relative to their genomic abundance. As shown in the Methods, this relationship can be derived explicitly within an extended mtDNA–RNA model. The use of scRNAseq therefore provides a practical approximation, but future applications to single-cell mtDNA sequencing data will be preferable whenever suitable datasets become available.

Using two stochastic models of the mitochondrial life cycle, we generated synthetic datasets that serve as a gold standard. Despite differing assumptions about mitochondrial regulation, ATP homoeostasis versus fixed copy number, both models produced nearly identical distributions of mutant accumulation times. This demonstrates that the detailed regulation of mitochondrial turnover is not critical for the macroscopic dynamics of mutant expansion. Crucially, only mutants with a selective replication advantage were able to achieve takeover, reinforcing the conclusion that drift alone is insufficient in short-lived species.

Beyond accumulation times, we analysed the distribution of unique mutant species per cell, a highly informative observable. Experimentally, post-mitotic cells are typically dominated by one or very few deletion species. Our simulations reproduced this pattern whenever a subset of mutants possessed a selection advantage. Remarkably, the number of unique mutants per cell followed an almost perfect Poisson distribution, characterized by a single parameter *λ*. This finding is nontrivial since the mitochondrial life cycle includes selection, competition, and degradation processes that could in principle distort such a simple statistical structure. The persistence of a Poisson distribution indicates that the stochastic generation of rare mutation events is the dominant determinant of mutant diversity, while selection primarily filters which of these mutants persist.

Systematic variation of model parameters revealed that λ depends linearly on the mutation probability and the fraction of advantageous mutations, but shows a nonlinear, approximately quadratic dependence on selection advantage and age. These scaling relationships provide additional mechanistic insight, suggesting that mutational input governs diversity in a proportional manner, whereas selection and time amplify early-arising mutants and suppress later competitors in a nonlinear fashion. The emergence of such simple mathematical relationships from a biologically complex system underscores the robustness of the underlying processes and suggests that similar patterns may be observable in real data.

To connect these insights to experimental measurements, we employed the Moran process as a tractable mathematical framework for modelling mutant dynamics^37–39^. The Moran model accurately reproduced the accumulation time distributions obtained from both stochastic simulations, even when the simulations violated some of the model’s underlying assumptions. We then demonstrated that the Moran process can be fitted directly to cross-sectional data, using summary statistics such as the fraction of cells exceeding different mutant thresholds. Applying global optimisation and parametric bootstrapping, we showed that key parameters like mutation probability, selection advantage, and fraction of advantageous mutants can be recovered with high accuracy and well-defined confidence intervals.

Importantly, we quantified how parameter uncertainty scales with sample size, demonstrating that confidence intervals derived from synthetic data provide a principled estimate of uncertainty when analysing real scRNAseq datasets. This is particularly relevant given that experimental studies often involve only a few thousand cells. The results further highlight that selection advantage is the most informative parameter for discriminating between competing hypotheses, as it directly reflects whether mutant accumulation is driven by drift or positive selection.

A limitation of the present framework concerns the identifiability of fracAdv, the fraction of deletion mutants that carry a selective advantage. Our bootstrap results indicate that this parameter becomes only partially identifiable when its true value is close to the upper boundary of the admissible range, such that values like fracAdv=0.8 and fracAdv=1 are difficult to distinguish reliably. This should not be interpreted as a general failure of the method, since the problem is much weaker in parameter regimes where fracAdv lies well within the interior of the allowed range. Rather, it reflects a structural limitation of the cross-sectional observables used here. In future work, this limitation could be alleviated by incorporating deletion breakpoint information, because under our hypothesis, selectively advantaged deletions should cluster in specific hotspot regions of the mitochondrial genome. Such location data would provide an independent empirical constraint on fracAdv and should improve its estimation.

In summary, we have developed and validated a comprehensive framework for extracting quantitative information about mtDNA mutant dynamics from cross-sectional single-cell data. By integrating stochastic simulations, analysis of mutant diversity, Moran process modelling, and robust parameter inference, we provide a unified approach to study mtDNA deletion accumulation. This framework not only reconciles several previously conflicting observations but also offers concrete, testable predictions for experimental datasets. Application of this approach to large-scale single-cell transcriptomic resources is essential for testing these predictions in vivo. Accordingly, a companion manuscript applying the framework to experimental data from the Tabula Muris Senis project^43^ has been submitted separately (see also our preprint at^42^), providing a direct analysis of mtDNA mutant accumulation dynamics in ageing mouse tissues and extending the methodological results presented here.

## Methods

### Stochastic simulation of mitochondrial life-cycle

The stochastic simulations were implemented in Java and are based on an individual-based representation of mitochondrial DNA molecules within a single post-mitotic cell. Each simulation trajectory represents the lifetime dynamics of mtDNA populations under replication, degradation, and mutation, corresponding to the stochastic analogue of the deterministic ODE models described in the main text.

At any time point, the cellular mtDNA population is represented as a set of mtDNA ‘types’, where each type corresponds to a distinct mtDNA species (wild type or a specific deletion mutant) together with its current copy number. Initially, all mtDNA molecules are wild type. When a mutation occurs during replication of a wild-type mtDNA molecule, a new mutant lineage is created and thereafter tracked as a separate mtDNA type within that cell. In this sense, mutant lineages are defined at the level of individual mtDNA genomes, not at the level of whole mitochondria or whole cells. Each newly arising mutant type is assumed to be unique and is assigned either no selective advantage or a selective replication advantage, depending on the model parameters.

The parameter *mutProb* denotes the mutation probability per replication event of a single wild-type mtDNA molecule. Thus, whenever a wild-type mtDNA molecule replicates, the daughter molecule becomes a deletion mutant with probability *mutProb*, otherwise it remains wild type. Mutated mtDNA molecules are not allowed to mutate further in the present implementation. This is a simplifying assumption, motivated by the fact that the quantities analysed here, in particular the accumulation-time distributions and the fraction of takeover events, are dominated by whether or not a selectively advantaged mutant lineage appears. In biological terms, this is also consistent with our hypothesis that selectively advantaged deletion mutants owe their advantage to loss of components of a negative transcriptional feedback system. Such a loss cannot be reversed by a subsequent deletion. A secondary mutation that would convert a previously non-advantaged mutant into an advantaged one is in principle possible, but test calculations showed no appreciable effect on the observables considered in the present study.

Time is discretized in steps of one hour. At each step, two stochastic processes are applied, representing degradation and replication. Degradation is modelled as a binomial process, where for each mtDNA type the number of molecules lost in a time step is drawn from a binomial distribution with parameters given by the current copy number and a degradation probability derived from the specified half-life. Replication is likewise treated stochastically: for each mtDNA type, the expected number of replication events is determined based on the ODEs described in the main text. The actual number of replication events is then drawn from a binomial distribution. For wild-type mtDNA, each replication event can give rise to a deletion mutant with a specified mutation probability; newly arising mutants are assigned their replication rate at birth and are tracked as separate types thereafter. All degradation, replication, and mutation events are sampled using optimised binomial random number generators based on the Marsaglia–Tsang–Wang algorithm, as implemented in the Apache Commons RNG library.

Simulation output is recorded to a data file at fixed reporting intervals specified by the user (e.g. 30 days). At each reporting point, the current mtDNA composition is stored, including the copy numbers of all mtDNA types and their associated replication rates. A trajectory is considered “taken over” when the fraction of mutant mtDNA exceeds a predefined threshold.

### Using the Moran process to model accumulation times

To model the spread of mtDNA mutants, we employed the Moran process^37–39^, a classical stochastic model from population genetics that is well suited for describing how a new variant spreads in a finite population of constant size. In the Moran model, each step consists of two events, the random selection of one individual that will reproduce and another is randomly selected to die, so that the population size remains fixed. The probability that a particular type is chosen for reproduction depends on its relative fitness. As a result, a mutant with a selective advantage is slightly more likely to increase in number, whereas a neutral or disadvantaged mutant may still drift but is more prone to extinction. Over time, the system can only end in one of two absorbing states, the mutants either go extinct or they reach fixation, fully replacing the wild-type population.

We model a cell containing N (e.g. 1000) mtDNA molecules, starting with a single mutant and the remainder wild type. Types differ by a multiplicative replication advantage, with fitness values given by ${f}_{\alpha }=1+{s}_{\alpha }$ (wild type *s*_α_ = 0). If the mutant carries a selection advantage, this bias alters the birth–death dynamics in its favour. The central quantity of interest is the distribution of times required for the mutant population to expand to dominance. Because the Moran process is a discrete-time Markov chain with well-defined transition probabilities between states {*i* = 0, 1, …, N}, it is possible to calculate this distribution exactly. We used a recursive first-step analysis:

6 $${f}_{i}\left(t\right)={p}_{i}^{+}{f}_{i+1}\left(t-1\right)+{p}_{i}^{-}{f}_{i-1}\left(t-1\right)+{p}_{i}^{0}{f}_{i}\left(t-1\right),{\rm{for}}\;{t}\ge 1,$$

with boundary conditions:

- ${f}_{N}(0)=1$ (if we start with mutants only, fixation time is 0 with probability 1),
- ${f}_{i}(0)=0$ (cannot be fixed at time 0 if not already in state N).

The probability of fixation at time t from state *i* can be written in terms of the probabilities of moving to neighbouring states at the first step and then fixing in *t* − *1* steps from there. With suitable boundary conditions, the full distribution can be built iteratively. Alternative formulations, such as spectral decompositions of the transition matrix^44^, provide equivalent results and confirm that fixation time distributions are typically broad and right-skewed.

### Using the Moran process to calculate probability distributions

To obtain probability distributions rather than single stochastic trajectories, we write the forward master equation for the count state *m* = (*m*₁,…,*m*_K_) of *K* mutant classes and wild-type ${m}_{0}=N-\mathop{\sum }\limits_{i=1}^{K}{m}_{i}$. The only allowed transitions are *m* → *m* + e_γ_ − e_δ_, representing one birth of type γ and one death of type δ. The transition probability for a Moran step (from state **m**) involving one birth of type γ and one death of type δ is given by:

7 $$T[(\gamma ,\delta )|{\boldsymbol{m}}]=\left(\mathop{\sum }\limits_{\alpha =0}^{K}\frac{{f}_{\alpha }{m}_{\alpha }}{{\sum }_{{\mathcal{l}}}{f}_{{\mathcal{l}}}{m}_{{\mathcal{l}}}}\cdot {u}_{\alpha \to \gamma }\right)\times \frac{{m}_{\delta }}{N},$$

This yields a sparse, time-homogeneous Markov kernel on the feasible lattice $\{{\boldsymbol{m}}:{m}_{i}\ge 0,\mathop{\sum }\limits_{i}{m}_{i}\le N\}$. Starting from a pure wild-type initial condition, we propagate the probability tensor *P*_t_(*m*) forward by one step with this kernel and sample it at the reporting times.

To map from real time to Moran steps, we use the organelle replication rate ${r}_{b}=\mathrm{ln}2/\mathrm{halfL}$, so that one ‘Moran step’ corresponds to one replication and degradation event in the cell. Thus over Δ*t* days, the expected number of steps is $N{r}_{b}\Delta t$. Computationally, this enables efficient vectorized updates on the grid. This exact, mechanistic construction produces time-resolved probability distributions for multiple mutant classes with distinct selection advantages, parameterised in physical units.

For parameter estimation, we minimised the sum of squared differences between the Moran predictions and the empirical curves across all time points. For optimisation we employed the Python library “fcmaes” (https://github.com/dietmarwo/fast-cma-es), specifically its Differential Evolution (DE) algorithm. DE is a population-based, gradient-free optimisation method, well suited for nonlinear, multimodal optimisation problems. To obtain parameter estimates together with statistically meaningful uncertainty measures, we employed parametric bootstrapping. Specifically, we generated 40 independent synthetic data sets using stochastic simulations of the Moran model with known parameters. Each synthetic data set was then analysed using the same parameter fitting pipeline. From the resulting empirical distributions of parameter estimates, 95% confidence intervals (CI) were obtained as the 2.5th and 97.5th percentiles.

Because the mapping from Moran steps to physical time depends on the assumed mtDNA half-life and mtDNA copy number, we additionally performed a sensitivity analysis to assess how robust parameter estimation is to misspecification of these quantities. Synthetic data were generated with the baseline parameter values used throughout the study (*N* = 1000, halfL = 10 days), but the fitting procedure was then repeated under alternative assumptions for either mtDNA half-life or mtDNA copy number while leaving all other aspects of the analysis unchanged. Specifically, we repeated the fits using halfL=20 and 30 days, and using *N* = 500 and 1500 molecules per cell. For each condition, 40 independent fits were performed (with the exception of 22 fits for *N* = 1500) and the resulting parameter estimates and fit qualities were summarised by their median values. This analysis makes it possible to quantify how strongly the inferred values of *mutProb*, *selAdv*, and *fracAdv* depend on imperfect prior knowledge of turnover and copy number (Tables 4 and 5).

**Table 4:** Sensitivity of fitted parameter values to misspecification of mtDNA half-life

halfL	mutProb median	selAdv median	fracAdv median	SSQ median
10	6.750E-06	0.10865	0.86365	9.30754E-06
20	8.23E-06	0.25475	0.6348	9.74427E-05
30	9.46E-06	0.4134	0.56755	0.000191618

Shown are median values from 40 independent fits.

Synthetic data were generated with halfL=10 days and N = 1000, but fitting was repeated assuming different values of halfDNA.

**Table 5:** Sensitivity of fitted parameter values to misspecification of mtDNA copy number

N	mutProb median	selAdv median	fracAdv median	SSQ median
500	1.47E-05	0.09	0.7247	0.000101225
1000	6.75E-06	0.10865	0.86365	9.30754E-06
1500	4.27E-06	0.1146	0.83	3.56E-05

days and *N* = 1000, but fitting was repeated assuming different values of *N*. Shown are median values from 40 independent fits for *N* = 500 and 1000 and 22 independent fits for *N* = 1500 (due to limitations of compute resources).

Synthetic data were generated with halfL = 10 days and N = 1000, but fitting was repeated assuming different values of N. Shown are median values from 40 independent fits for N = 500 and 1000 and 22 independent fits for N = 1500 (due to limitations of compute resources).

As expected, the quality of the fits deteriorated when the assumed values used for inference differed substantially from those used to generate the synthetic data. In particular, misspecification of mtDNA half-life led to systematic upward shifts in the estimated values of *mutProb* and *selAdv*, whereas misspecification of the copy number had more modest effects with especially *selAdv* remaining largely uneffected. These results show that the inference framework is conditionally identifiable, i.e. it reliably estimates *mutProb*, *selAdv*, and *fracAdv* only for given assumptions about mtDNA copy number and turnover. In practice, however, this does not represent an insurmountable limitation, because approximate values for these quantities can often be obtained from the literature, and mtDNA copy number could in principle be measured directly in future applications based on single-cell DNA sequencing.

### Relating RNA-based heteroplasmy to DNA-based heteroplasmy

In the present study, mutant accumulation is inferred from single-cell RNA sequencing (scRNAseq) data rather than from single-cell DNA sequencing. This is motivated by the fact that deletions in the mitochondrial genome are also reflected at the transcript level. However, because the selective advantage postulated for certain deletion mutants is assumed to arise from impaired transcriptional feedback, the relationship between RNA-based heteroplasmy and DNA-based heteroplasmy is not expected to be exactly one-to-one. In particular, mutants with a selective advantage may be somewhat overrepresented at the RNA level relative to their abundance at the DNA level. To quantify this effect, we considered an extended version of Model-2 in which the dynamics of wild-type and mutant mtDNA are coupled to the dynamics of their corresponding RNA pools.

The extended model consists of four ordinary differential equations describing the time evolution of wild-type mtDNA (DNAwt), mutant mtDNA (DNAmt), wild-type RNA (RNAwt), and mutant RNA (RNAmt):

8 $$\frac{{dDN\,Awt}}{{dt}}={k}_{{rep}}\frac{{DN\,Awt\cdot RN\,Awt}}{{DN\,Aw\cdot tRN\,Awt}+{DN\,Amt\cdot RN\,Amt}}({N}_{{dna}}-{DN\,Awt}-{DN\,Amt})-{DN\,Awt}\frac{\mathrm{ln}2}{{half\,DNA}}$$

9 $$\frac{{dDN\,Amt}}{{dt}}={k}_{{rep}}\frac{{DN\,Amt\cdot RN\,Amt}}{{DN\,Awt\cdot RN\,Awt}+{DN\,Amt\cdot RN\,Amt}}({N}_{{dna}}-{DN\,Awt}-{DN\,Amt})-{DN\,Amt}\frac{\mathrm{ln}2}{{half\,DNA}}$$

10 $$\frac{{dRN\,Awt}}{{dt}}={k}_{{tr}}({N}_{{rna}}-{RN\,Awt})-{RN\,Awt}\frac{\mathrm{ln}2}{{half\,RNA}}$$

11 $$\frac{{dRN\,Amt}}{{dt}}={k}_{{tr}}({N}_{{rna}}-{k}_{i}{RN\,Amt})-{RN\,Amt}\frac{\mathrm{ln}2}{{half\,RNA}}$$

The shape of the first two equations follows directly from the assumptions of Model-2, but now makes explicit that replication initiation depends on the local availability of RNA-derived primers. The total number of mtDNA molecules is limited by the hard upper bound N_dna_, and replication events are distributed between wild-type and mutant genomes according to their relative contributions to the total pool of replication-priming RNA. Thus, the probability that the next replication event occurs in the wild-type class is proportional to *DNAwt⋅RNAwt*, while the corresponding probability for the mutant class is proportional to *DNAmt⋅RNAmt*. Both wild-type and mutant mtDNAs are assumed to have the same half-life, *halfDNA*.

The last two equations describe the dynamics of the RNA pools. For wild-type mtDNA, transcription is assumed to be subject to negative feedback, represented by the factor

(*N*_rna_ − RNAwt). In this formulation, *N*_rna_ defines the scale at which transcription becomes progressively inhibited as the amount of wild-type RNA increases. For mutant mtDNA, the same general structure is retained, but the negative feedback is weakened by the factor *ki*, where 0 *<* *ki* ≤ 1. The parameter *ki* therefore represents the residual strength of transcriptional inhibition in the mutant relative to wild type. A value of *ki* = 1 corresponds to a mutant with the same transcriptional regulation as wild type and thus no selective advantage, whereas smaller values of *ki* correspond to progressively weaker feedback and hence increased transcriptional activity of the mutant. We do not assume complete absence of feedback (*ki* = 0), because even mutants that have lost the local cis-acting feedback elements may still be exposed to indirect limitation through diffusion of functional products or through other cellular constraints on transcription.

This four-equation system provides a mechanistic interpretation of the selection advantage parameter *selAdv* used in the reduced two-variable model. In that reduced model, the mutant class is assigned an explicit replication bias of *1+selAdv* relative to wild type. In the present extended model, the corresponding bias emerges from the ratio of mutant to wild-type RNA levels. At equilibrium of the fast RNA subsystem, one obtains

12 $${RN\,Aw}{t}^{*}=\frac{{k}_{{tr}}{N}_{{rna}}}{{k}_{{tr}}+{\mathrm{ln}}2/{half\,RNA}}{\mathrm{and}}\,{{\mathrm{RN}}\,{\rm{Amt}}}^{* }=\frac{{k}_{{tr}}{N}_{{rna}}}{{k}_{i}{k}_{{tr}}+{\mathrm{ln}}2/half\,RNA}$$

The ratio of mutant to wild-type RNA at equilibrium is therefore

13 $$\frac{{RN\,Am}{t}^{* }}{{RN\,Aw}{t}^{* }}=\frac{{k}_{{tr}}+{\mathrm{ln}}2/{half\,RNA}}{{k}_{i}{k}_{{tr}}+{\mathrm{ln}}2/{half\,RNA}}.$$

If RNA dynamics are fast compared with mtDNA turnover, and if transcription dominates RNA degradation, i.e. k_tr_≫ln2/halfRNA, this expression simplifies to

14 $$\frac{{RN\,Am}{t}^{* }}{{RN\,Aw}{t}^{* }}\approx \frac{1}{{k}_{i}}.$$

Comparison with the original two-equation formulation then shows that the effective mutant replication bias is given by

15 $$1+{selAdv}\approx \frac{{RN\,Amt}}{{RN\,Awt}}\approx \frac{1}{{k}_{i}},\mathrm{so}\,\mathrm{that}\,selAdv\approx \frac{1}{{k}_{i}}-1$$

Thus, the phenomenological parameter *selAdv* of the reduced model can be interpreted mechanistically in terms of the degree to which transcriptional feedback is weakened in the mutant. The same framework can be used to derive the relationship between the fraction of mutant mtDNA molecules and the fraction of mutant RNA molecules. Let

16 $${f}_{{DNA}}=\frac{{DN\,Amt}}{{DN\,Awt}+{DN\,Amt}}$$

denote the mutant fraction at the DNA level, and let f_RNA_ denote the corresponding mutant fraction at the RNA level. If each mtDNA molecule rapidly establishes its own local RNA pool, then the total contribution of mutant genomes to the cellular RNA population is proportional to DNAmt⋅RNAmt, whereas the contribution of wild-type genomes is proportional to DNAwt⋅RNAwt. It follows that

17 $${f}_{{RNA}}=\frac{{DN\,Amt\cdot RN\,Amt}}{{DN\,Amt\cdot RN\,Amt}+{DN\,Awt\cdot RN\,Awt}}.$$

Dividing numerator and denominator by RNAmt gives

18 $${f}_{{RNA}}=\frac{{DN\,Amt}}{{DN\,Amt}+({RN\,Awt}/{RN\,Amt}){DN\,Awt}}.$$

Under the quasi-steady-state approximation discussed above, it holds in good approximation that

19 $$\frac{{RN\,Awt}}{{RN\,Amt}}\approx {k}_{i},{\rm{so}}\;{\rm{that}}\,{f}_{{RNA}}=\frac{{DN\,Amt}}{{DN\,Amt}+{k}_{i}{DN\,Awt}}.$$

Expressing DNAwt and DNAmt in terms of f_DNA_ yields

20 $${f}_{{RNA}}=\frac{{f}_{{DNA}}}{{f}_{{DNA}}+{k}_{i}(1-{f}_{{DNA}})},\mathrm{or}\,{\mathrm{equivalently}},\,{f}_{{RNA}}=\frac{{f}_{{DNA}}}{{k}_{i}+(1-{k}_{i}){f}_{{DNA}}}$$

This expression shows that RNA-based heteroplasmy systematically exceeds DNA-based heteroplasmy whenever *ki* < 1, i.e. whenever mutant transcription is less strongly inhibited than wild-type transcription. At the same time, the deviation is bounded and depends in a simple and predictable way on the parameter *ki*. This relationship is illustrated in Fig. S1 for ki = 0.333, 0.4, and 0.5, corresponding approximately to effective selection advantages of *selAdv*≈2, 1.5, and 1, respectively. The plot demonstrates that RNA heteroplasmy indeed overestimates DNA heteroplasmy, but that the deviation is moderate over a broad range of values relevant for the present study.

Taken together, these considerations justify the use of scRNAseq data as a practical first approximation to the underlying mtDNA heteroplasmy, while also making clear that RNA-based measurements are not an exact surrogate for direct DNA-based measurements. This limitation should be borne in mind when interpreting fitted parameter values and provides a further motivation for future application of the framework to single-cell mtDNA sequencing datasets.

## Supplementary information

Figure S1

Supplementary Figure

## Data Availability

Data and computer code used for this work is available via our GitLab repository at https://gitlab.uni-rostock.de/IBIMA/MitoMutantAccTimes1.

## Code availability

Data and computer code used for this work is available via our GitLab repository at https://gitlab.uni-rostock.de/IBIMA/MitoMutantAccTimes1.
