# Supplementary figures and images for: Inferring accumulation times of mitochondrial DNA deletion mutants from cross-sectional single-cell data: methodological framework and validation
Source: NPJ Aging. 2026 Jun 16;12(1):83. doi: 10.1038/s41514-026-00431-4 (PMC13272806; doi:10.1038/s41514-026-00431-4)

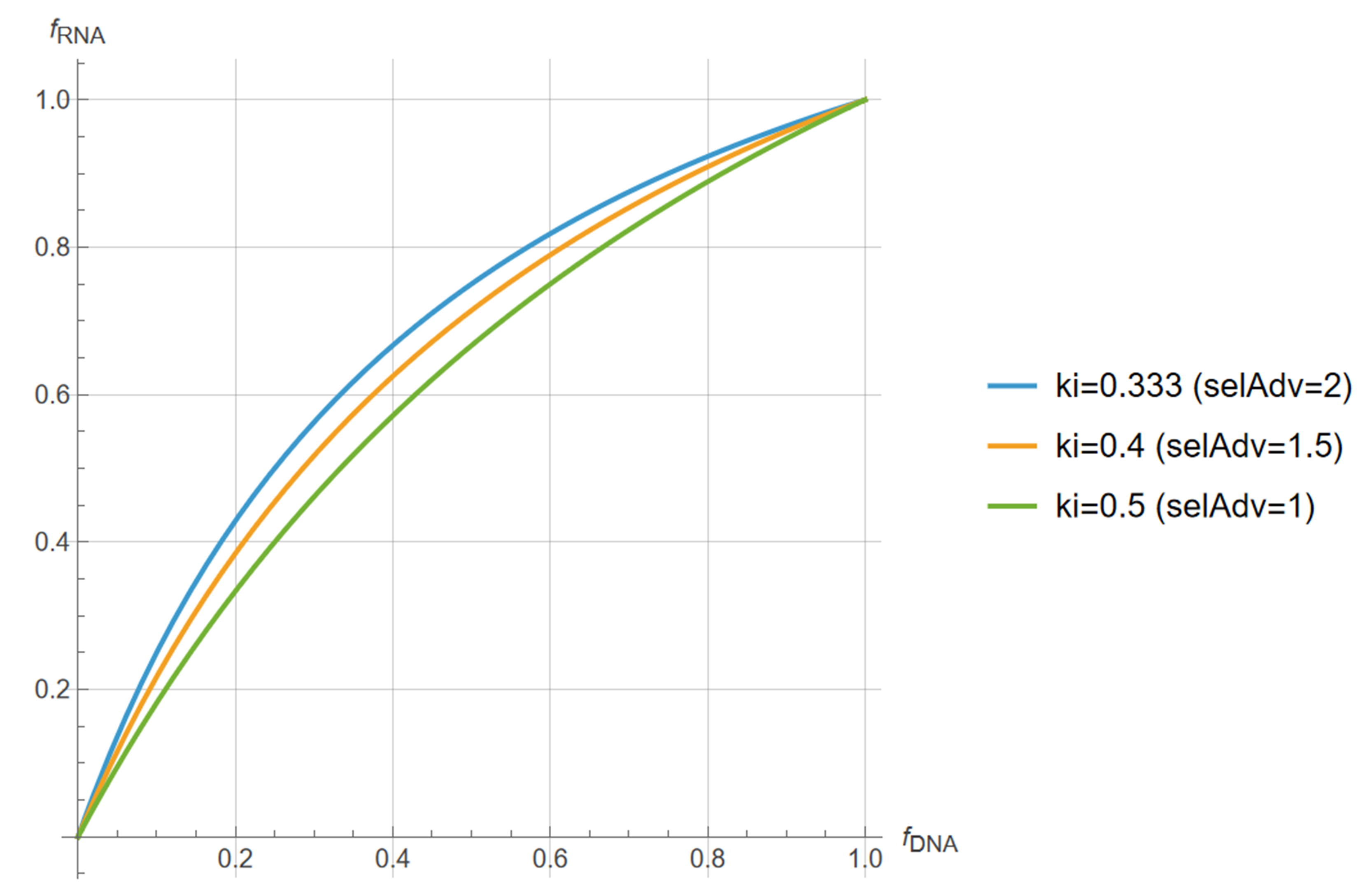

Supplement: Supplementary file 1 — Figure S1 [file 41514_2026_431_MOESM1_ESM.tif]
