# Supplementary material for: Inferring accumulation times of mitochondrial DNA deletion mutants from cross-sectional single-cell data: methodological framework and validation
Source: NPJ Aging. 2026 Jun 16;12(1):83. doi: 10.1038/s41514-026-00431-4 (PMC13272806; doi:10.1038/s41514-026-00431-4)
Supplement: Supplementary file 2 — Supplementary Figure [file 41514_2026_431_MOESM2_ESM.docx]

Fig. S1 Relationship between mutant RNA fraction (f_RNA_) and mutant DNA fraction (f_DNA_) for different values of the residual feedback parameter *ki*. Curves are shown for *ki*=0.333, 0.4, and 0.5, which correspond approximately to effective selection advantages of selAdv≈2, 1.5, and 1, respectively. For *ki*<1, RNA-based heteroplasmy exceeds DNA-based heteroplasmy, but the deviation remains bounded.
